# Supplementary material for: A Likelihood Approach for Real-Time Calibration of Stochastic Compartmental Epidemic Models
Source: PLoS Comput Biol. 2017 Jan 17;13(1):e1005257. doi: 10.1371/journal.pcbi.1005257 (PMC5240920; doi:10.1371/journal.pcbi.1005257)
Supplement: S1 File — (TAR.GZ) [file pcbi.1005257.s014.tar.gz › HSPH_Online-SI-Revision/Additions/EnKF08/n100-extreme-SEITR_noisy/n100-extreme-SEITR_noisy_EnKF_table-8.pdf]

1	R0	{1.38654, 9.85565}	{2.09043, 8.10475}	{{0.371522, 3.01244}, {3., 13.041}}	{91.6665, 43.4823}	inf	369.	5980.98	{3178.48, 12794.7}	1047.98	0.907552	newly	644.29	5215.52	{1334., 17625.}	709.498	0.685117	Reff	1.36736	0.386703	{0.062852, 0.709709}	75.3198	ar	92379.	191811.	{179610., 239920.}	107.635
2	R0	{2.36629, 12.2029}	{2.30128, 9.72553}	{{0.924616, 1.99163}, {3., 17.1033}}	{29.4794, 42.7223}	inf	10.	25143.	{7044.5, 45190.3}	179493.	3.14869	newly	20.	27539.3	{4940., 63069.}	137597.	2.94942	Reff	2.3656	1.37267	{0.301009, 2.22208}	45.919	ar	99022.	123225.	{73303., 173430.}	35.1546
3	R0	{2.32253, 12.836}	{1.71873, 8.94123}	{{0.389575, 2.00079}, {3., 16.7918}}	{43.3035, 40.8507}	inf	18.	10498.8	{2836.07, 19268.3}	38784.6	2.42921	newly	26.	17979.8	{2482., 56411.}	69053.	2.52168	Reff	2.32139	1.43158	{0.273104, 2.85519}	47.188	ar	98997.	73046.4	{17613., 109552.}	38.2596
4	R0	{1.79075, 11.2058}	{2.31021, 9.6511}	{{1.57969, 4.05989}, {3., 17.0983}}	{44.4374, 37.6056}	inf	68.	28922.1	{9784.65, 61074.1}	29716.6	2.37507	newly	88.	25230.6	{6701., 52236.}	28571.1	2.32013	Reff	1.78676	1.30919	{0.348312, 2.22042}	36.9327	ar	96920.	129498.	{87674., 172115.}	39.7181
5	R0	{2.26952, 9.62371}	{1.81874, 7.7527}	{{0.215207, 2.00079}, {3., 15.86}}	{40.3879, 52.727}	inf	2.	7639.26	{1694.25, 13635.4}	152685.	3.05418	newly	5.	20956.2	{2042., 69163.}	419023.	3.26109	Reff	2.26934	1.62125	{0.215204, 2.97847}	41.8801	ar	98881.	73596.8	{15758., 108307.}	31.2667
6	R0	{1.57191, 6.89004}	{2.23583, 10.0939}	{{0.989911, 2.10871}, {3., 16.3418}}	{59.4774, 65.6666}	inf	11.	17582.6	{5559.13, 39708.9}	146422.	3.04476	newly	22.	20194.4	{4724., 45125.}	91692.6	2.76945	Reff	1.57147	1.69199	{0.339346, 2.75207}	41.9417	ar	95046.	127454.	{56364., 210104.}	46.8181
7	R0	{1.99226, 17.2339}	{2.39721, 7.45989}	{{1.45361, 3.38682}, {3., 10.3007}}	{40.3514, 56.7138}	inf	713.	2299.37	{1294.5, 1938.24}	120.881	0.190861	newly	648.	2440.52	{705., 2478.}	276.648	0.296459	Reff	1.94486	0.486474	{0.01561, 0.590925}	75.1538	ar	97992.	182280.	{174365., 182569.}	86.0155
8	R0	{2.63369, 15.1879}	{2.2798, 10.7725}	{{1.00378, 7.65882}, {3., 17.0606}}	{27.6238, 36.6592}	inf	35.	31377.3	{6341.2, 82350.1}	60240.9	2.50138	newly	41.	22969.3	{4716., 41467.}	55922.6	2.55785	Reff	2.6309	1.19651	{0.164174, 2.07276}	55.2274	ar	99451.	130384.	{72178., 184570.}	40.7257
9	R0	{2.53271, 9.90552}	{1.98329, 7.23686}	{{0.663261, 2.00079}, {3., 11.5986}}	{34.7246, 44.5027}	inf	2.	8910.82	{2837.07, 19411.8}	445441.	3.5107	newly	8.	25223.2	{2330., 75289.}	315190.	3.17324	Reff	2.53258	1.75154	{0.654667, 2.971}	36.7104	ar	99355.	82463.7	{25374., 109326.}	32.1067
10	R0	{2.48791, 18.9003}	{2.56077, 7.35768}	{{2.15963, 4.22697}, {3., 14.7367}}	{18.0661, 61.1073}	inf	205.	25777.9	{8419.45, 52371.}	8188.71	1.77489	newly	173.	25865.	{6120., 59624.}	14850.9	2.01213	Reff	2.47216	0.974975	{0.250726, 2.18558}	60.5618	ar	99291.	163798.	{119909., 239252.}	70.1706
11	R0	{2.84414, 18.4765}	{2.53028, 4.98131}	{{1.7665, 1.10871}, {3., 12.8343}}	{17.2827, 73.0397}	inf	68.	21610.5	{4371.16, 53088.3}	20881.1	2.15747	newly	76.	25835.5	{4513., 72959.}	33894.1	2.31429	Reff	2.83774	0.633186	{0.288291, 1.14098}	77.687	ar	99654.	147867.	{88662., 247699.}	55.1929
12	R0	{2.29369, 14.622}	{2.52458, 7.49294}	{{2.15615, 4.25314}, {3., 14.7793}}	{17.6314, 50.7392}	inf	100.	21613.5	{4399.79, 47180.6}	15228.7	2.04354	newly	114.	24337.6	{5209., 59651.}	21248.8	2.13093	Reff	2.28683	1.04286	{0.407399, 2.18425}	54.3972	ar	98982.	157217.	{106221., 230929.}	62.1687
13	R0	{2.73413, 16.7529}	{2.35553, 5.42329}	{{0.848488, 2.17239}, {3., 10.1327}}	{24.7382, 67.752}	inf	62.	29509.3	{9373.5, 59629.1}	37732.4	2.43545	newly	67.	41323.	{7425., 104055.}	61576.1	2.58917	Reff	2.72932	1.07823	{0.273248, 2.17338}	60.4944	ar	99543.	138022.	{70956., 199585.}	50.224
14	R0	{1.79554, 16.5943}	{2.1415, 9.45835}	{{0.418629, 2.91064}, {3., 13.1498}}	{59.1417, 43.0024}	inf	652.	5286.31	{2700.96, 4059.3}	454.121	0.547664	newly	541.	3938.69	{1137., 12433.}	628.039	0.610102	Reff	1.75512	0.403967	{0.0790682, 0.634771}	76.9835	ar	96821.	181929.	{172463., 212834.}	87.9028
15	R0	{1.91429, 13.7771}	{2.43401, 6.30306}	{{1.4667, 3.4159}, {3., 9.83803}}	{40.6983, 54.2497}	inf	266.	3049.97	{445.898, 5273.42}	706.383	0.632426	newly	278.	4193.98	{267., 4409.}	1411.91	0.751385	Reff	1.89691	0.566262	{0.197921, 1.37042}	70.7907	ar	97541.	191029.	{177610., 245494.}	95.8453
16	R0	{1.42094, 10.5871}	{2.23372, 4.03911}	{{1.09713, 2.65265}, {3., 4.94494}}	{72.5111, 61.8487}	inf	260.	12963.	{2346.26, 31036.2}	3315.02	1.38215	newly	379.	14047.4	{4313., 36574.}	3609.96	1.38994	Reff	1.40736	0.34432	{0.0412977, 0.808022}	75.5343	ar	93044.	154063.	{91649., 194276.}	70.299
17	R0	{1.45515, 6.16466}	{1.87356, 7.05197}	{{0.232799, 2.00079}, {3., 14.9852}}	{67.0254, 78.3898}	inf	5.	10972.	{2049.24, 27501.2}	365632.	3.37474	newly	11.	26131.1	{3055., 67685.}	237456.	3.08069	Reff	1.45502	1.62721	{0.220993, 2.76589}	53.2068	ar	93254.	80183.	{16287., 108938.}	35.5588
18	R0	{2.85669, 13.2416}	{2.28211, 8.39393}	{{0.894113, 2.11084}, {3., 12.879}}	{24.9268, 40.5577}	inf	3.	16973.3	{5494.59, 42070.}	154203.	3.07103	newly	8.	30270.2	{4705., 89678.}	378277.	3.34881	Reff	2.85618	1.76107	{0.320072, 2.99997}	40.0534	ar	99644.	133488.	{52080., 200116.}	49.7459
19	R0	{2.56417, 17.9558}	{2.16362, 4.16871}	{{1.16269, 2.64591}, {3., 4.99244}}	{31.5096, 76.7835}	inf	91.	12980.2	{4545.93, 23982.1}	8274.35	1.78176	newly	91.	15121.2	{6503., 44021.}	16516.7	2.07147	Reff	2.55604	0.453956	{0.0514498, 1.15213}	82.2399	ar	99389.	153073.	{82895., 193341.}	61.8191
20	R0	{2.27517, 11.1864}	{2.12695, 9.1941}	{{0.783866, 2.10869}, {3., 13.6202}}	{31.3146, 35.2885}	inf	8.	13993.4	{4737.78, 26501.4}	87358.5	2.83477	newly	17.	22263.7	{3476., 71444.}	130863.	2.8839	Reff	2.27446	1.72276	{0.457873, 2.7351}	31.7315	ar	98894.	126239.	{59121., 198017.}	44.3958
21	R0	{2.35735, 10.4735}	{1.85046, 7.85223}	{{0.196957, 2.00079}, {3., 16.4484}}	{41.5847, 46.9875}	inf	5.	9703.	{1564.91, 23802.2}	193960.	3.09715	newly	5.	19289.5	{2224., 56025.}	385690.	3.28316	Reff	2.35707	1.57199	{0.196676, 2.86656}	42.9146	ar	99036.	74980.8	{13637., 107837.}	36.2464
22	R0	{1.79551, 11.9179}	{2.52681, 7.00639}	{{2.14923, 4.14832}, {3., 14.7527}}	{42.7121, 47.1609}	inf	143.	25076.	{5541.36, 55042.9}	11146.1	1.88949	newly	205.	29337.9	{5882., 72170.}	14211.7	1.9382	Reff	1.78616	1.08275	{0.410587, 2.18509}	49.8848	ar	97033.	162418.	{66075., 235925.}	76.333
23	R0	{1.23545, 6.31687}	{2.32316, 6.23797}	{{0.848263, 2.17245}, {3., 10.1206}}	{101.414, 46.4084}	inf	66.	30774.9	{11269.3, 59650.3}	24920.2	2.30562	newly	213.	39260.3	{10838., 85989.}	18332.1	2.12305	Reff	1.23161	1.11527	{0.482538, 1.67428}	29.8491	ar	88739.	149439.	{93714., 196430.}	73.1308
24	R0	{2.24089, 10.1789}	{2.29694, 9.11541}	{{1.29904, 2.10875}, {3., 14.6935}}	{27.9886, 38.3938}	inf	4.	17934.3	{4648.96, 41680.5}	298805.	3.33208	newly	6.	25476.5	{3066., 77953.}	424508.	3.39062	Reff	2.24062	1.71986	{0.386032, 2.61299}	30.8573	ar	98868.	127343.	{71706., 187171.}	40.1795
25	R0	{1.57298, 10.2607}	{2.40388, 5.25618}	{{1.76449, 1.17854}, {3., 12.8934}}	{54.2301, 60.0223}	inf	107.	24805.6	{5142.66, 54826.6}	17370.6	2.05864	newly	146.	27992.9	{2383., 73087.}	19074.7	2.03745	Reff	1.56721	0.59182	{0.162029, 1.36516}	64.0287	ar	94957.	152004.	{70657., 248117.}	69.0998
26	R0	{1.2154, 8.2576}	{2.02342, 4.88862}	{{0.052166, 0.909737}, {3., 5.7654}}	{114.314, 51.7153}	inf	374.	4450.55	{275.72, 12055.5}	869.809	0.726171	newly	559.	4543.62	{231., 10431.}	735.045	0.655005	Reff	1.19834	0.250087	{0.00783009, 0.44488}	79.1305	ar	88421.	148339.	{61726., 172353.}	81.5897
27	R0	{2.22841, 18.7983}	{1.99137, 4.08163}	{{0.0892991, 0.911475}, {3., 5.75156}}	{45.1569, 78.2872}	inf	579.	4717.79	{663.502, 14009.4}	469.98	0.603329	newly	487.	5483.93	{460., 15461.}	1035.1	0.806892	Reff	2.1875	0.301535	{0.0129302, 0.441849}	86.6868	ar	98756.	165756.	{145445., 231306.}	78.9622
28	R0	{1.27778, 5.36767}	{2.0288, 8.7555}	{{0.62999, 2.00079}, {3., 15.0178}}	{80.5365, 100.858}	inf	15.	11085.1	{3015.12, 30588.}	61483.6	2.63513	newly	37.	22676.6	{2437., 72734.}	61188.1	2.47519	Reff	1.27717	1.69947	{0.534591, 2.86709}	64.3024	ar	89843.	89901.6	{22171., 110428.}	38.4506
29	R0	{2.38042, 15.8901}	{2.16753, 5.45222}	{{0.850038, 2.17481}, {3., 7.74307}}	{32.485, 65.6879}	inf	75.	28360.9	{8136.79, 52658.7}	22408.6	2.23214	newly	93.	36856.6	{12121., 69439.}	39530.8	2.44423	Reff	2.37421	1.0641	{0.353576, 1.74114}	55.6141	ar	99112.	135002.	{72845., 191560.}	46.9105
30	R0	{2.53188, 12.7607}	{1.99683, 8.56283}	{{0.452935, 2.00079}, {3., 16.1279}}	{35.4106, 43.1467}	inf	1.	9638.96	{2259.25, 22316.6}	192679.	3.1156	newly	3.	22448.9	{1544., 77135.}	748195.	3.51219	Reff	2.5317	1.75148	{0.396249, 2.97063}	37.4491	ar	99317.	81780.1	{16086., 109356.}	35.2922
31	R0	{1.24482, 10.5471}	{2.03944, 9.1934}	{{0.357752, 2.89011}, {3., 12.9106}}	{105.103, 36.83}	inf	904.	4832.97	{3088.53, 3392.14}	280.563	0.458846	newly	1087.	3850.1	{1325., 9530.}	255.273	0.36676	Reff	1.19689	0.434565	{0.0667036, 0.639143}	66.3705	ar	89097.	186579.	{177477., 219957.}	109.421
32	R0	{2.30446, 17.9607}	{2.48355, 6.49108}	{{2.15895, 4.25633}, {3., 11.5004}}	{17.7843, 63.8595}	inf	288.	22955.8	{5317.93, 34740.4}	5276.08	1.57284	newly	254.	29881.9	{4084., 72807.}	11664.5	1.84546	Reff	2.28427	1.0743	{0.329695, 2.05206}	52.9699	ar	99028.	153343.	{93326., 222352.}	60.186
33	R0	{2.31579, 11.8236}	{1.86611, 8.48535}	{{0.364094, 2.00079}, {3., 15.9364}}	{40.3778, 45.1208}	inf	22.	8913.02	{1314.49, 22959.5}	31737.1	2.24157	newly	33.	22184.7	{801., 71774.}	67128.1	2.30305	Reff	2.31442	1.60058	{0.227043, 2.73435}	41.1324	ar	99012.	73890.2	{12535., 108627.}	34.821
34	R0	{1.6678, 10.6744}	{2.47132, 6.96043}	{{2.16093, 4.13474}, {3., 9.96737}}	{50.0064, 43.8012}	inf	174.	22125.9	{2578.36, 54330.2}	9042.94	1.81714	newly	267.	28957.3	{3314., 72948.}	10745.4	1.81422	Reff	1.65861	1.15642	{0.394857, 2.18547}	49.3297	ar	95809.	167451.	{88200., 247554.}	82.8005
35	R0	{1.65476, 7.07559}	{1.82629, 7.91976}	{{0.357111, 2.00079}, {3., 15.6729}}	{57.9818, 68.6465}	inf	1.	8147.2	{3987.34, 13153.4}	101740.	2.873	newly	10.	21396.7	{2364., 75997.}	213868.	2.95939	Reff	1.65458	1.55587	{0.3063, 2.92988}	52.3796	ar	95746.	73900.9	{16279., 107158.}	35.915
36	R0	{2.71807, 18.5959}	{2.49044, 7.28616}	{{2.15928, 4.21247}, {3., 14.7381}}	{17.0876, 60.9411}	inf	74.	22375.3	{8936.55, 36793.4}	25040.8	2.27741	newly	61.	23962.6	{6296., 59543.}	39183.	2.42402	Reff	2.71258	0.952192	{0.319675, 2.10905}	64.8972	ar	99540.	148546.	{82868., 230880.}	56.8782
37	R0	{2.6202, 12.7552}	{2.05118, 5.84942}	{{0.847592, 2.17245}, {3., 10.0977}}	{33.023, 56.4778}	inf	2.	26422.1	{6505.39, 49196.7}	377359.	3.44652	newly	7.	39224.5	{4561., 106074.}	560250.	3.52484	Reff	2.61991	1.0691	{0.266939, 2.17367}	59.7341	ar	99426.	141893.	{100317., 197417.}	45.5641
38	R0	{1.47672, 9.81651}	{2.1569, 4.36643}	{{1.17085, 2.64597}, {3., 5.0309}}	{64.1855, 56.8913}	inf	196.	15131.7	{8683.51, 50688.4}	4457.75	1.52668	newly	329.	15627.3	{6752., 48123.}	4649.94	1.52667	Reff	1.46491	0.342132	{0.100564, 0.619829}	76.6448	ar	93815.	164906.	{86246., 255898.}	81.1638
39	R0	{1.90004, 12.3313}	{2.56964, 7.41131}	{{2.1565, 4.36517}, {3., 14.7482}}	{38.4838, 46.0961}	inf	121.	20452.8	{4465.88, 47363.8}	11791.2	1.91897	newly	167.	22407.5	{4278., 57625.}	13317.7	1.91076	Reff	1.89271	0.971634	{0.27146, 2.05163}	52.2933	ar	97413.	148313.	{84247., 226410.}	59.2732
40	R0	{2.64218, 18.5237}	{2.20198, 5.86267}	{{0.847679, 2.17243}, {3., 10.0985}}	{30.7448, 68.3505}	inf	208.	30300.4	{7822.49, 70087.6}	10760.3	1.9058	newly	187.	38731.6	{7660., 85347.}	20612.1	2.14708	Reff	2.62693	1.0968	{0.264168, 1.72059}	58.2478	ar	99474.	139117.	{88727., 200400.}	46.0104
41	R0	{1.6945, 7.17996}	{1.85922, 9.28516}	{{0.569303, 2.00079}, {3., 17.7598}}	{50.952, 76.8164}	inf	3.	9060.61	{2420.45, 17790.8}	181112.	3.092	newly	9.	19496.7	{1893., 64732.}	216529.	2.96199	Reff	1.69433	1.6461	{0.320422, 2.85805}	46.6818	ar	96332.	79172.7	{16000., 106084.}	33.0011
42	R0	{1.84559, 18.0074}	{2.40425, 6.8548}	{{1.4635, 3.38717}, {3., 9.94677}}	{41.8405, 61.9334}	inf	1046.	2886.86	{1282.84, 4201.01}	111.235	0.168371	newly	870.	3548.58	{741., 5346.}	318.322	0.288658	Reff	1.78061	0.559214	{0.200629, 0.718994}	71.0361	ar	97358.	193058.	{182256., 203280.}	98.2965
43	R0	{2.24965, 17.0361}	{2.51192, 7.22448}	{{2.16533, 4.20731}, {3., 14.7376}}	{19.6234, 57.5931}	inf	172.	22770.4	{6682.82, 31999.4}	9310.91	1.8492	newly	161.	25556.	{6330., 72327.}	15773.3	2.01339	Reff	2.23721	1.02135	{0.296905, 2.18152}	54.3473	ar	98770.	155168.	{81936., 238902.}	65.4687
44	R0	{2.54324, 15.7967}	{2.50267, 7.257}	{{2.16518, 4.28719}, {3., 14.7193}}	{17.8044, 54.06}	inf	68.	23176.3	{6403.05, 41709.6}	19710.6	2.15525	newly	107.	25833.6	{4405., 71038.}	24045.4	2.20102	Reff	2.53714	1.0868	{0.331104, 2.18128}	57.1644	ar	99354.	150266.	{66464., 222648.}	61.6076
45	R0	{2.54943, 14.8032}	{2.17203, 10.2267}	{{0.937949, 7.67099}, {3., 17.0717}}	{29.8536, 38.5759}	inf	40.	23557.6	{5019.67, 40351.1}	41229.1	2.49359	newly	25.	20506.	{4738., 28805.}	40912.1	2.44715	Reff	2.54637	1.32691	{0.288255, 2.22016}	48.9309	ar	99380.	126595.	{81333., 171405.}	35.0713
46	R0	{2.37755, 12.6461}	{1.96378, 7.8021}	{{0.357216, 2.00079}, {3., 14.6138}}	{37.3008, 45.7068}	inf	8.	9059.82	{2638.32, 19957.5}	56523.8	2.56925	newly	26.	26505.3	{2371., 82656.}	101843.	2.61469	Reff	2.37696	1.76666	{0.293455, 2.99997}	39.421	ar	99096.	82059.1	{17170., 109268.}	33.8749
47	R0	{1.69279, 11.6636}	{2.31376, 5.33977}	{{1.75528, 1.17193}, {3., 12.8847}}	{36.6831, 59.6286}	inf	138.	26478.2	{4734.23, 55946.6}	13008.	1.98658	newly	167.	29942.7	{4563., 67486.}	17829.7	2.07086	Reff	1.68417	0.62085	{0.288697, 1.32606}	63.9489	ar	96214.	166195.	{81008., 254126.}	80.2325
48	R0	{1.82402, 10.0038}	{2.29061, 10.4574}	{{0.947964, 4.02194}, {3., 17.107}}	{43.5925, 39.926}	inf	25.	26373.3	{3741.47, 54751.9}	79819.1	2.69958	newly	39.	23942.5	{2569., 60526.}	61293.2	2.52873	Reff	1.82252	1.24548	{0.279402, 2.2221}	41.154	ar	97076.	127353.	{77403., 183764.}	41.3389
49	R0	{1.6424, 11.1667}	{2.47431, 7.20982}	{{2.15923, 4.23672}, {3., 14.7703}}	{54.2657, 41.722}	inf	267.	26566.	{6747.15, 57206.2}	6677.05	1.67888	newly	380.	26494.2	{6372., 70756.}	6872.16	1.67731	Reff	1.62686	0.997205	{0.330887, 2.01633}	50.1756	ar	95456.	151794.	{83690., 231799.}	66.1089
50	R0	{2.05638, 14.3048}	{2.1644, 5.69899}	{{0.839323, 2.1732}, {3., 10.1128}}	{41.5552, 60.8906}	inf	167.	28349.1	{8189.02, 54349.4}	12727.6	1.9697	newly	176.	36674.4	{10241., 70128.}	20737.7	2.12753	Reff	2.0461	1.00591	{0.316027, 1.51727}	51.8532	ar	98194.	135157.	{81806., 199072.}	47.3821
